# No speed dating please! Patterns of social preference in male and female house mice

**DOI:** 10.1186/s12983-017-0224-y

**Published:** 2017-07-24

**Authors:** Miriam Linnenbrink, Sophie von Merten

**Affiliations:** Max- Planck Institute for Evolutionary Biology, Plön, Germany

**Keywords:** Social preference, Decision making process, Mate choice, *Mus musculus domesticus*, MHC, Familial imprinting

## Abstract

**Background:**

In many animal species, interactions between individuals of different sex often occur in the context of courtship and mating. During these interactions, a specific mating partner can be chosen. By discriminating potential mates according to specific characteristics, individuals can increase their evolutionary fitness in terms of reproduction and offspring survival. In this study, we monitored the partner preference behaviour of female and male wild house mice (*Mus musculus domesticus*) from populations in Germany (G) and France (F) in a controlled cage setup for 5 days and six nights. We analysed the effects of individual factors (e.g. population origin and sex) on the strength of preference (selectivity), as well as dyadic factors (e.g. neutral genetic distance and major histocompatibility complex (MHC) dissimilarity) that direct partner preferences.

**Results:**

Selectivity was stronger in mice with a pure population background than mixed individuals. Furthermore, female mice with a father from the German population had stronger selectivity than other mice. In this group, we found a preference for partners with a larger dissimilarity of their father’s and their partner’s MHC, as assessed by sequencing the H2-Eß locus. In all mice, selectivity followed a clear temporal pattern: it was low in the beginning and reached its maximum only after a whole day in the experiment. After two days, mice seemed to have chosen their preferred partner, as this choice was stable for the remaining four days in the experiment.

**Conclusions:**

Our study supports earlier findings that mate choice behaviour in wild mice can be paternally influenced. In our study, preference seems to be potentially associated with paternal MHC distance. To explain this, we propose familial imprinting as the most probable process for information transfer from father to offspring during the offspring’s early phase of life, which possibly influences its future partner preferences. Furthermore, our experiments show that preferences can change after the first day of encounter, which implies that extended observation times might be required to obtain results that allow a valid ecological interpretation.

**Electronic supplementary material:**

The online version of this article (doi:10.1186/s12983-017-0224-y) contains supplementary material, which is available to authorized users.

## Background

In social animals, individuals interact with each other in a broad range of different situations. Interactions between individuals of different sex often occur in the context of courtship, pair bonding, and mating. A preference for some possible social partners over others can ultimately lead to mate choice. The evolution of mate choice is assumed to be driven by several mechanisms [1], such as preferences for direct or indirect phenotypic benefits and genetic correlations between mating preferences and preferred traits [2].

Selective mating is meant to increase the evolutionary fitness of individuals in terms of reproduction and offspring survival [1, 3–6]. Consequently, different mating strategies have evolved. Assortative mating results from the reproduction of phenotypically or genotypically matching mates and promotes population differentiation [7, 8]. In contrast, disassortative mating occurs when individuals prefer dissimilar mates compared to neutral expectations [9–12]. The latter strategy maintains or even increases genetic variability and counteracts possible disadvantages due to inbreeding depression (e.g. [13]).

An important basis for mate choice behaviour lies in the evolution of mechanisms to recognize conspecifics’ characteristics and thus identify potential mates. In mice (*Mus musculus sp.), the* recognition of individuals, families, and populations is mainly regulated by two sensory systems: olfaction and vocalisation. Chemical signals used are volatiles (i.e. pheromones), peptides (e.g. of the major histocompatibility complex, MHC), and proteins (e.g. major urinary proteins) [14–16]. Acoustic communication occurs via ultrasonic vocalisation [16–18].

The MHC (officially termed “H2” in mice [19]) is a highly polymorphic gene complex that encodes many proteins with key roles in the adaptive immune system. Since Yamazaki and colleagues [20] detected MHC-related mating preferences in laboratory mice, many studies have reported an influence of MHC loci on mate choice in nearly all classes of vertebrates [21]. Potential reasons for MHC being involved in mate discrimination are kin recognition and the enhancement of the offspring’s immune competence, which occurs by increasing either MHC diversity or dissimilarity by choosing a compatible mate [22]. An important pre-requisite for MHC-based mate choice in mice is the ability to identify and discriminate potential partners based on MHC alleles. Several studies have analysed the influence of MHC on partner choice for house mice. They mostly support the hypothesis of disassortative mating [23, 24] and raised evidence for familial imprinting [23, 25]. Familial imprinting is the non-genetical transmission (i.e. learning is involved) of preferences (e.g. preference for food, home area or mates (see Immelmann [26] for review) during the early phase of life from mostly one parent as reference to its offspring.

Wild mice offer a perfect model system to study the behavioural and genetic basis of mate choice, since they are still far more natural than common laboratory mouse strains in both aspects. Due to the vast amount of studies on lab mice, numerous genetic tools are available and can be also applied for studies in wild mice. Examples of such study populations are two originally wild caught populations of *M. m. domesticus* (one population from France and one from Germany), which separated about 3000 years ago. This separation is reflected in the divergence of the nuclear genome and gene expression differences [27–29], as well as in ultrasonic vocalisation [30]. Montero and colleagues [31] studied the degree of mutual mate recognition according to population origin under semi-natural conditions and identified complex mating patterns in these two populations. Assortative mating according to population background was only observed when mice of the single populations could get familiar with each other before individuals of both populations had the chance to meet. Further, the mating patterns observed were based on paternally influenced mate preferences, such that mice with a father from the French population preferred mating with a partner from that population, individuals fathered by a German male preferred mating with an individual from the German population. The authors suggested genomic or familial imprinting as being involved.

The aim of the present study is to reveal possible factors that determine the mating patterns found by Montero et al. [31] in more detail. We studied the exact two mouse populations mentioned to shed light on the evolution of mate choice in the early phase of population differentiation. By using a controlled cage setup, we were able to follow single (focal) individuals during their decision making processes and analyse the persistency of their choices. To offer the same possible mates as in the study by Montero and colleagues [31], focal mice were allowed to choose between four partners of either the same or different population origins (France and Germany) or reciprocal crosses of both. Focal mice were females and males of the same four genotypes. We aimed to confirm the findings of preferences for paternally matching population backgrounds. In order to do so we investigated three different aspects of mouse behaviour in our setup, (i) general activity of the focal mice, (ii) their degree of selectivity (i.e. strength of preference independent of its direction) and underlying temporal changes when several potential partners where presented, and (iii) factors correlating with the direction of preference, including the effect of MHC on preference behaviour, which might possibly serve as evidence for MHC-driven partner preference.

## Methods

### Animals

The mice used for this study originate from two *M. m. domesticus* populations. The ancestors of the experimental individuals were originally caught in France in the Massif Central (2005) and in Germany around Cologne/Bonn (2006), and were kept in the mouse facility at the MPI Plön under outbred conditions to maintain genetic and behavioural variability. At the time of the experiment, mice from the French population were in the 8th, mice from the German population in the 6th and 7th generation. A specific breeding has been set up for this experiment and we obtained experimental mice from 15 breeding pairs. Pure offspring of both populations (“German” GG and “French” FF) and reciprocal crosses between mice of the two populations (“mixed individuals”), either with a mother from the German and a father from the French population (GF) or with a mother from the French and a father from the German population (FG) have been bred. All mice were kept and raised under standard conditions together with both parents until weaning at the age of four weeks. Female and male offspring were kept separated after weaning to ensure no sexual experience before the experiment. We had a balanced system of experimental mice: six females and males per genotype (GG, FF, GF, FG), which results in a total number of 48 mice in the behavioural experiment. For genetic analyses, we additionally included the parents of the focal mice and some siblings that had not been used in the behavioural experiment, resulting in genetic samples from 76 individuals. All information on mice including population background, sex, microsatellite data and experimental information can be found in Additional file 1: Table S1.

### Experimental setup and procedure

**Fig. 1:**
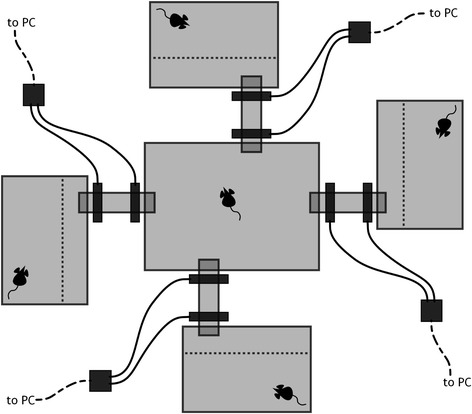
Schematic top view of the experimental setup. One central cage is connected via Plexiglas tubes with four satellite cages. Satellite cages are divided by a metal grid (*dotted lines*) to prevent mating of the focal mouse (with access to the central cage and the smaller inner parts of the satellite cages) and the four satellite mice (with access only to the larger outer part of their respective satellite cage). Each tube is fitted with a double RFID ring antennae system, all connected to a PC to record the movements of the the focal mouse, which is equipped with an RFID tag. Food, water and shelter were provided for all mice, but are not shown in the figure for clarity

The experimental setup consisted of five standard macrolon cages (Techniplast). One central cage (40.5 × 28.0 × 20.0 cm) and four Type III satellite cages, which were connected via Plexiglas tubes to each one of the four sides of the central cage (Fig. 1). Each Plexiglas connection was equipped with two RFID ring antennae (TSE Industries Inc.), one close to the central cage and one close to the respective satellite cage. Each mouse was equipped with an RFID tag (Iso FDX-B, Planet ID), which is read every time the mouse passes one of the antennae.

The satellite cages were divided into two parts by a metal grid. In each of the four satellite cages, a so-called satellite mouse was placed in the larger, outer part. The smaller inner part could be accessed by the so-called focal individual from the central cage, in which it was placed at the beginning of the experiment. This setup allowed the focal mouse to move between the central and all outer cages. Through the separation mice could interact (i.e. via smell and vocalisation), but copulation and thus, from an animal ethical viewpoint unwanted, offspring was prevented. Water and food as well as bedding were provided for all mice ad libitum, for the satellite mice in their part of the respective cage, for the focal mouse in the central cage. The order a mouse participated in the experiment (first as focal vs. first as satellite mouse, which we randomised as much as possible within genotype and equal between genotypes) had no effect on their behaviour in the experiment (Additional file 1: Table S1).

Each group of satellite mice, consisting of four mice of similar sex and differing genotype (FF, GG, FG, and GF), formed a satellite-quad. The mice in each quad stayed the same over experiments to ensure a comparable measure of preference across cage systems. Each quad participated four times as satellite mice, one time for each of the four possible genotypes of focal mice.

Each run of the experiment was started by placing the focal and satellite mice in their respective cages and starting the computer program monitoring the RFID antennae. Each experiment ran over five days and six nights, always starting around 16:00 h on a Thursday and finishing the following Wednesday around 8:00 h. The rooms were set with a dark-light cycle of 12:12 h with lights on at 7:00 and off at 19:00 h, both for the breeding before the experiments, as well as during the experiments.

We chose such a comparatively long duration of the experiment as we were interested in the decision making process. Further, in this time frame, each female mouse can be assumed to have concluded at least one full oestrus cycle [32]. We decided against a daily control of the females’ oestrus state as a two week pilot experiment showed a very high sensitivity to any disturbances in the experimental room. Females also usually enter oestrus synchronised as soon as they perceive certain pheromones of a nearby male [33], which was the case in our study.

### Behavioural data

For each focal individual a text-file was generated with time stamps for every antenna read, and the identification number of the respective antenna. By this, we could gain information about the number of antenna reads as a proxy for activity. Using a self-written script in R [34] we calculated the duration of time spent in the four satellite cages, which served a proxy for preference behaviour of the focal mouse.

### Genetic data

#### Microsatellite genotyping and analysis

To determine if genetic distance is an important factor of partner preference we chose 13 unlinked microsatellite markers published by Teschke et al. [27]. These markers are Chr3_24R, Chr16_21R, CHr12_05R, Chr10_45R, Chr01_25R, Chr17_09R, Chr05_45R, Chr13_22R, Chr19_08R, Chr14_16R, Chr09_20R, Chr01_23, and Chr02_02R. Forward primers were labelled with FAM or HEX, and PCR was performed using 5 ng/μl DNA template together with the Multiplex PCR kit (QIAGEN). After processing PCR products with HiDi formamide and 500 ROX size ROX standard, samples were run on an ABI 3730 sequencer (Applied Biosystems). Raw alleles have been called using GeneMapper 4.0 (Applied Biosystems). The proportion of shared alleles [35] and the pairwise genetic distance (Cavalli-Sforza distance; in the present paper referred to as CAS) between all individuals were calculated with the program MSA [36] and visualized using MEGA 6 [37]. As a paternal influence on partner choice was proposed by Montero and colleagues [31], we also included the genetic distance from the mother and father of the focal mouse to each of the satellite mice (referred to as CASmat or CASpat, respectively).

#### Sequencing and analysis of the H2-Eß locus

We chose one locus of the MHC Class II complex (H2-Eß) for Sanger Sequencing and, more specifically, decided to focus on Exon 2 as this exon is known for determining the antigen binding groove and thus might be most directly involved in pathogen resistance and thus interesting for partner choice. Primers used for PCR and sequencing are: Forward 5’CGG GCA TCT TGT CGG CAG AGA AGA AG 3′ and Reverse 5’CAC CGT GGT TCC GCC CCA GCC ACC 3′. Sequences were edited manually with Seqman (included in DNASTAR, Inc., Madison, USA) and aligned with the algorithm Clustal-W [38], included in the program MEGA 6 [37]. The phase of diploid sequences was estimated following Stephens and colleagues [39, 40], implemented in DNASp [41]. To assess whether MHC-dissimilarity between two potential mating partners were important, the number of amino acid differences per site (p-distance) between sequences of the focal individual to the four satellite individuals was calculated, as well as the p-distance between the mother and father of the focal individual to each of the satellite mice (in this paper referred to as MHC, MHCmat and MHCpat, respectively). The latter has been done, to identify a possible influence of the parents on the offspring’s choice. Furthermore, we addressed the question if MHC-diversity of the potential mate is influencing mate choice. Therefore, we again calculated the number of amino acid differences per site (p-distance) between the two haplotypes within each satellite individual. Individual H2-Eß sequences have been submitted to Dryad.

### Statistical analysis

#### Patterns of activity

To estimate the activity patterns of focal mice, we analysed the number of antenna reads per hour for each focal mouse. We tested for changes in activity over time using a generalised linear model (with Poisson error distribution, fitting to our count data) with experimental day, genotype and sex as fixed factors, and activity per hour as response variable. We used square-root transformed data to improve distribution of residuals (checked with QQ-plot and Shapiro-Wilk test: W = 0.9948, *p* = 0.3522). For the analysis of temporal behavioural patterns, we had to exclude one of the focal mice, as during the run of one individual (a GF male) the recording was stopped unintentionally after three days due to power failure.

#### Selectivity of focal mice

To analyse the selectivity (i.e. the strength of preference that focal mice show for some satellite mice over others, independent on the direction of preference), we calculated a selectivity index SI with the formula SI = SD/SD(max), where SD is the standard deviation of the four proportional durations (in %) that the respective focal mouse spent in each of the four satellite cages, and SD(max) is the maximal standard deviation theoretically possible, which is in our case (4 possibilities) 50. The resulting SI values range from 0 (25% of time in each of the four satellite cages = no selectivity) to 1 (100% of time in one of the four satellite cages, no time in the three others = highest selectivity). SI was calculated in ten minute intervals for each focal mouse to get measures for the change of selectivity over time, and over the whole period to get a measure of overall selectivity for each individual.

To calculate which factors might influence the selectivity, we applied a generalised linear model with the overall SI per focal mouse as response variable. We used the best fitting error distribution, which was a beta-distribution. The tested factors were the sex of the focal individual, its maternal and paternal population background, and whether it is of pure or mixed population background. The t- haplotype is a selfish genetic element that has previously been shown to affect mate choice in house mice. Even though some experimental animals carried the t-haplotype, we could not detect any influence of t-haplotype on selectivity and thus retained all individuals in the analysis, irrespective of their t-haplotype status, and included the t-haplotype status of the focal mouse as a random effect in the model testing for selectivity.

#### Factors correlating with preference

To test if the direction of preference of focal mice depends on characteristics of the satellite mice and/or dyadic factors between focal and satellite mice (e.g. genetic distance), we applied a generalised linear mixed effects model (function glmmadmb from the R package glmmADMB which allows for a beta error distribution, which was the best fit for our data). The proportion of duration spent in each of the satellite cages served as response variable, and identity of the focal mouse, satellite-quad and t-haploytpe of the satellite mouse as random factors. The following characteristics of satellite mice served as fixed factors: maternal and paternal population background (i.e. F or G), information whether the satellite individual is of pure or mixed population origin (i.e. FF and GG vs. FG and GF), information whether the population background is matching with that of the focal individual (i.e. paternal matching (e.g. F**G** chooses G**G**), maternal matching (e.g. **F**G chooses **F**F), exact match *(*e.g. **GF** chooses **GF**), no match (e.g. GF chooses FG), MHC diversity (p-distance of the two amino acid alleles of the satellite mouse), MHC (p-distances of each satellite mouse to the focal individual), MHCmat and MHCpat (p-distances of each satellite mouse to the focal individual’s mother or father, respectively), CAS (genetic distances based on microsatellites between each satellite mouse and the focal individual), CASmat and CASpat (genetic distance of each satellite mouse to the focal individual’s mother or father, respectively). We further included cage position as an additional factor to test if the preference of focal mice was influenced by the position of the cage inside the experimental room. During experiments, we had already aimed to minimise a possible position effect by randomising the position of satellite mice (by genotype) over trials.

In a second step, following the results from the analysis of selectivity (see Table 1) and the results from the models analysing the direction of preference (see Table 2), we performed correlations to analyse the influence of MHCpat on the direction of preference, separated by the groups that differ in selectivity. We calculated Spearman rank correlation coefficients between the duration spent in each of the satellite cages and the factor MHCpat, grouped by the type of population background (pure vs. mixed), and by sex and paternal population background. We used square-root transformed data to improve distribution of residuals (checked with a QQ-plot and Shapiro-Wilk test: W = 0.9818, *p* = 0.0915).

**Table 1:** Influence of focal mouse characteristics on selectivity

Factor (characteristics of focal mice)	df	X^2^	p
Maternal population background	1	0.6581	0.41723
Paternal population background	1	2.9311	0.08689
Sex	1	0.6974	0.40365
**Pure or mixed population background**	**1**	**5.3001**	**0.02132**
First as focal or first as satellite mouse	1	0.0154	0.90127
Maternal population background: Sex	1	0.0173	0.89533
**Paternal population background**: **Sex**	**1**	**5.4789**	**0.01925**

Presented are the results of all fixed effects of the generalised linear model, degrees of freedom (df), Chi-Square values (X^2^) and *p*-values. T-haplotype status was included as random effect. Significant results are printed in bold

**Table 2:** Effect of different parameters on the direction of preference

Factor (characteristics of satellite mice)	df	X^2^	P
Maternal population background	1	0.0346	0.8525
Paternal population background	1	0.9560	0.3282
Pure or mixed population background	1	1.2095	0.2714
Relative matching population background	3	1.9556	0.5817
MHC diversity	1	0.0001	0.9906
MHC distance to the focal mouse	1	0.2943	0.5875
MHC distance to the mother of the focal mouse	1	0.7897	0.3742
**MHC distance to the father of the focal mouse**	**1**	**4.3466**	**0.0371**
Genetic distance to the focal mouse	1	2.5150	0.1128
Genetic distance to the mother of the focal mouse	1	0.0001	0.9904
Genetic distance to the father of the focal mouse	1	0.0726	0.7876
Position of the respective satellite cage in the room	3	2.3073	0.5111

Presented are the results of all fixed effects of the generalised linear mixed model: degrees of freedom (df), Chi-Square values (X^2^) and *p*-values. Individual identity of focal mice, t-haplotype status and quad-number of satellite mice were included as random effects. Significant results are printed in bold

All statistical tests were carried out using R 2.14.1 [34].

#### Stability of preference

We used two different approaches to estimate the degree to which a preference is stable over time: First, we used the overall duration of time each focal mouse spent in the most preferred cage. Second, we determined in 10 min intervals the preferred cage for each focal mouse. We calculated how often this preference changes between cages, giving us the number of “preference blocks” as an estimator on how stable or unstable the choice of a focal mouse is: Few long preference blocks are a sign for a comparatively stable choice, many short preference blocks hint at a rather unstable choice. We further calculated how long the preference for the last preferred cage lasted, to estimate after how much time in the experiment on average the choice is established and how stable it is (see Additional file 2: Figure S1 for two examples). We calculated these preference block parameters over all mice and separated by sex.

Additionally we tested if the initial preferences (preferred satellite mouse after the first 10 min, 90 min and 24 h of the experiment) matched the final stable choice, using Chi-Square tests.

## Results

We monitored the partner preference behaviour of wild house mice (*M. m. domesticus*). Over five days and six nights, we allowed each of 24 males and 24 females to associate with their preferred partner among four individuals of opposite sex inside a controlled cage setup, which allowed sensory interaction via smell and vocalisation, but no full physical contact or mating (Fig. 1). Both the focal individuals and the potential partners differed in their genetic background with respect to population background (pure FF: population originating from France; pure GG: population originating from Germany; FG and GF: mixed population background, maternal origin given first). An allele-sharing tree shows genetic differentiation between FF and GG, even though the branch lengths are small and the level of differentiation is thus low (Additional file 3: Figure S2a). FG and GF individuals share alleles with both parent populations. When focusing on the MHC locus *H2-Eß*, this separation is not evident, and both populations share alleles (Additional file 3: Figure S2b). We used the duration that focal mice spent in each of the satellite cages as a proxy for their preference.

### Patterns of activity

Over the whole time in the setup, each mouse was registered 86 times per hour on average. The activity changed throughout the days of the experiment (LRT = 110.216, *p* < 0.001; Additional file 4: Figure S3), decreasing by an average of 19 antenna reads per hour on each day. Mice were most active during the first 24 h (including the complete first night), followed by a steep drop in activity and a steady but low, continuous decrease until the end of the experiment. This pattern is most likely due to habituation to the surroundings. Furthermore, we observed a daily rhythm in activity, with peaks occurring shortly after both lights-on and lights-off and highly reduced activity around mid-day (Additional file 4: Figure S3). A difference in activity could not be observed between sexes or genotypes (genotype: LRT = 4.767, *p* = 0.1897; sex: LRT = 0.609, *p* = 0.4351).

**Fig. 2:**
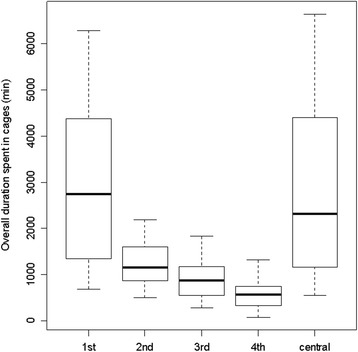
Duration of time spent in each of the four satellite cages and the central cage. The overall duration of time spent in the cage of the most preferred satellite mouse (1st) was higher than the duration of time spent in the cages of the three less preferred mice (2nd – 4th) and slightly higher than the duration of time spent in the central cage

When we compared the time spent in any of the satellite cages (“social time”) to the time spent in the central cage, we found that focal mice spent an average of 68.5% of their time close to another mouse rather than being alone in the central cage. The time that focal mice spent in the cage of the preferred mouse was approximately equal to the time spent in the central cage (central cage mean (sd) = 49.04 h (32.24 h), preferred cage: 49.31 h (27.55 h); Fig. 2). In contrast, most mice spent less time in each of the three non-preferred cages than the central cage (Fig. 2).

### Selectivity of focal mice

To analyse the selectivity of focal mice (i.e. the strength of preferences for some satellite mice over others, independent on the direction of preference), we calculated a selectivity index (SI) based on the time spent in satellite cages. SI ranges from 0 (no selectivity) to 1 (highest selectivity).

**Fig. 3:**
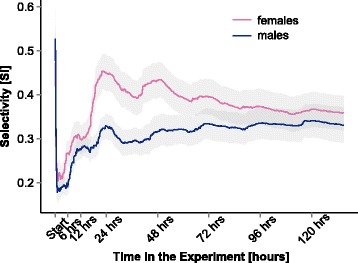
Temporal patterns of selectivity. Selectivity (given as SI, see Methods) measures the strength of preference. The average SI for males (*blue*) and females (*pink*) over the whole duration of the experiment is presented, including 95% confidence intervals

The selectivity changes over time (Fig. 3). In the first minutes of the experiment, the average values of SI are very high (SI_average(10min)_ = 0.50), followed by a steep drop. The lowest point occurs at about 90 min (duration: SI_average(90min)_ = 0.19). The high initial values of SI do not reflect selectivity itself, but rather the lack of time to explore other cages, which also explains the drop of selectivity when mice start to explore. After 90 min, SI increases again and reaches a maximum after 22.8 h among females (SI_average(max-females)_ = 0.45), while a local maximum occurs after 23.7 h among males (SI_average(local-max-males)_ = 0.33). The total maximum of males occurs only after 120.3 h (SI_average(max-males)_ = 0.34). Overall, females and males show a slight difference in their selectivity (SI_average(females)_ = 0.38 vs. SI_average(males)_ = 0.31). The temporal daily variation in SI reflects active vs. inactive phases of individuals and is congruent with the observed activity pattern (Additional file 4: Figure S3).

**Fig. 4:**
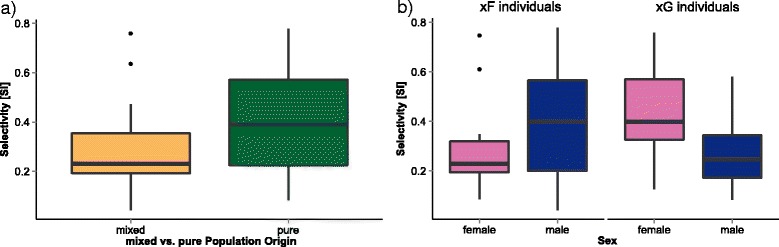
Three factors influencing selectivity. **a** Individuals with a pure population background (FF and GG, shown in *green*) choose stronger than those with a mixed population background (FG and GF, shown in *yellow*). **b** Paternal population background (F or G) and sex had a significant interactive effect on selectivity. xF individuals (mice with a father from the French population, irrespective of the mother’s population): xF males had a higher selectivity than xF females. xG individuals (mice with a father from the German population, irrespective of the mother’s population): xG females had a higher selectivity than xG males

Further, we aimed to identify factors involved in determining the selectivity of focal mice. Selectivity was significantly higher in mice with pure genotypes (FF, GG) as opposed to mixed individuals (FG, GF) (Fig. 4a, Table 1). Furthermore, the interaction between sex and paternal population background had a significant effect on selectivity (Fig. 4b, Table 1): males with a father from the French population showed higher selectivity than females with a father from the French population, while individuals with a father from the German population showed the opposite effect. Paternal population background and sex did not show significant effects on their own. Age and maternal population background also had no significant effects on selectivity.

### Factors correlating with preference

**Fig. 5:**
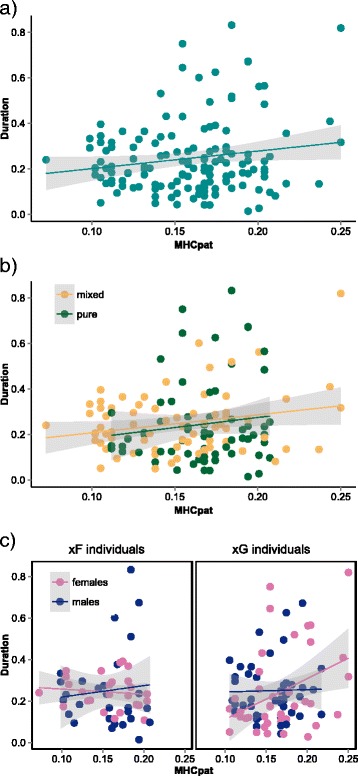
Correlation between the duration spent in satellite cages and the distance between the paternal MHC and the MHC of the respective satellite mouse (MHCpat). **a** Correlation between duration and MHCpat shown for all mice. **b** Separate correlations for focal individuals of pure or mixed population background. **c** Separate correlations for focal individuals depending on their sex and their paternal population background (i.e. if the father of the respective focal mouse belongs to the French or German population). For better visualisation of the data we used the linear model based smoothing function from the R package “ggplot2”

Using a generalised linear mixed effects model, we identified the paternal MHC as potential factor correlated with preference behaviour. The more distant the paternal MHC of the focal mouse is from that of the satellite mouse (MHCpat), the more time the focal mouse spent with the satellite mouse (Fig. 5a, Table 2). None of the other tested factors had any significant effects on preference.

As described before, we found significant differences in selectivity between pure and mixed mice, as well as a significant interactive difference in selectivity depending on the sex and the paternal population background of focal mice (see Results section, Selectivity of focal mice. Therefore, we tested for a correlation between MHCpat and the direction of preference using data separated according to these groups. We found no difference in the influence of MHCpat on preference between pure and mixed individuals (Fig. 5b, Table 3). When separated by sex and the paternal population background, MHCpat correlated with preference in only female mice with a father from the German population (xG females Fig. 5c, Table 3). Interestingly, the mean MHCpat was slightly higher (but not significantly) in females with a father from the German population (xG females, mean: 0.167, standard deviation: 0.039) compared to the other three groups (xF females: 0.154 ± 0.037; xG males: 0.157 ± 0.030; xF: 0.156 ± 0.034).

**Table 3:** Spearman correlation between the duration spent in satellite cages and the distance between the paternal MHC and the MHC of the respective satellite mouse

Group of data	R	T	df	P
All mice	0.1187	1.4042	138	0.1625
Separated by population background				
Pure population background	0.0907	0.7284	64	0.4690
Mixed population background	0.1896	1.6385	72	0.1057
Separated by sex and paternal population background				
xF females^a^	−0.1368	−0.6906	25	0.4962
**xG females**	**0.3656**	**2.4532**	**39**	**0.0187**
xF males	−0.0017	−0.0086	26	0.9932
xG males	−0.0017	−0.0110	42	0.9913

Values for spearman correlation are presented for all mice and separated by the groups differing in their strength of choice (i.e. mice with a pure population background vs. mice with a mixed population background; and mice differing in sex and their paternal population background). Significant results are printed in bold

^a^xF: individuals with a father from population F; xG: individuals with a father from population G

### Stability of preference

The overall proportion of time that each focal individual spent in its preferred satellite cage was 78.8% on average (minimum: 43.9%, maximum: 99.8%) of the total social time (the time spent in any of the satellite cages).

The mean number of preference blocks over all focal individuals is 7.5, and the duration of the last preference block is 66.7% on average. Results separated by females and males reflect the patterns of selectivity described above: females had a lower number of preference blocks on average (7.0) and a higher proportional duration of the last preference block (72.6) than males (number of blocks: 8.0; duration of last block: 60.8). This reflects a more stable preference in females than males. Considering the whole duration of the experiment (136 h), females did not change their preferences after an average of 37.3 h and males after 53.3 h.

The initial “preference” after 90 min (when selectivity was very low) and the final preference of focal mice matched in 13 of the 47 cases, which is not different from the matches expected by chance (11.75 of 47; Χ^2^(1) = 0.0034, *p* = 0.953). The preference after 24 h in the experiment matched the final preference in 23 of 47 cases, which is significantly higher than the expectation by chance (Χ^2^(1) = 5.713, *p* = 0.025). The “preference” after 10 min when the selectivity index SI was high and the final preference of focal mice only matched in 12 of the 47 cases, which is at chance level (Χ^2^(1) = 0.0000, *p* = 1). This confirms that our conjecture that high initial SI values do not reflect selectivity but the lack of time to explore all cages (see Patterns of activity).

## Discussion

We monitored the partner preference behaviour of wild house mice to characterize individual factors (e.g. population background) as well as dyadic factors (e.g. MHC dissimilarity between pairs of mice) correlating with preference behaviour and possibly mate choice. Moreover, to investigate the process that leads to the choice of a specific partner, we continuously tracked the behaviour of focal mice for nearly one week, which also assured that each female entered oestrus at least once.

### Patterns of activity

All mice exhibited similar activity patterns during the experiments, with reduced activity around midday and highest activity levels around “sun-down” and “sun-up”. Consistent with this activity pattern, we observed small daily changes in the strength of preference. As in most animals, daily activity patterns in mice are strongly influenced by light [42, 43], but cage enrichment, feeding schedule, and social factors also play a role [44, 45]. Our mice were most likely mainly entrained to the artificial day-night schedule maintained in our keeping facilities, but they also could have been influenced by social factors such as the activity of the four satellite mice. In line with this activity pattern, the duration that the focal mice spent in satellite cages peaked at regular intervals during midday (the time of lowest activity), when the focal mice were resting in one of the satellite cages with a chosen satellite mouse instead of being alone in the central cage. This resulted in the seemingly increased selectivity during midday.

### Decision making process in partner preference

To our knowledge, our study is the first to test the degree to which individuals prefer social partners over others, how this selectivity varies over an extended period of time, and at which time a stable preference is established. Most experiments on partner preference and mate choice have been based on short tests and usually last less than 10 min (e.g. [5, 46–49]). However, we chose to run our experiment for five days and six nights. Thonhauser et al. [50] used a long-term setup of 18 days to investigate the mate choice behaviour of female mice, but they only recorded the position of the focal mouse once per day. They were thus likely not able to follow the complete behavioural patterns, as some of the patterns we found changed on an hourly basis, especially in the beginning of the experiment. Our results clearly show a temporal change in selectivity over the course of the experiment. This change over time is consistent in all mice and might be part of a decision making process. Also Manser and colleagues [51] used RFID technique to monitor preference behaviour over several days. They did, however, not analyse their data with respect to temporal changes of selectivity. Instead, their analysis was based on the total time focal mice spent with the potential partners, comparable to our analysis on the direction of preference.

Selectivity values were low after the first minutes, as focal mice had just started to explore the new environment and to meet all four satellite mice. After 10 min, only half of the individuals had visited all satellite cages, and only after 90 min had all of them visited all satellite cages and thus met all possible companions. At this time, selectivity started to increase and peaked after about 24 h, and this maximum was more pronounced in females (see section Selectivity depends on sex and population background). At this time, the mice seemed to have explored their environment sufficiently to preferentially remain near one of the satellite mice. Indeed, final preferences were established and relatively constant after about 37.3 (females) and 53.3 h (males).

The oestrus cycle of female mice might potentially have an influence on selectivity, with a possibly increased selectivity in both sexes when entering oestrus. We did not control for oestrus and can thus not exclude that the change in oestrus status might have influenced selectivity in both sexes. The females of this study were housed without males before the onset of the experiment. Females housed in this way often enter an anoestrus state and only come into oestrus about three to four days after introduction of a male [52]. As selectivity in our experiment reached its peak after 24 h, the females have most likely not yet been in oestrus, which would make an influence of oestrous on selectivity in our study rather unlikely.

In only about one-quarter of the focal mice, the final preference of social partner was the same as the initial choice, which is what is expected by chance. We conclude that the aparent “choice” at the beginning of the experiment is differentiated from the final stable preference after about two days and might just be an artefact resulting from a lack of time to assess all given possibilities. The stability in preference for a given partner is in line with findings by Montero and colleagues [31], who detected a high degree of mate fidelity in a mate choice experiment under semi-natural conditions.

These results clearly indicate that the duration of the experiment can influence the measured selectivity and preference behaviour. Apart from possible influences of the oestrus cycle, a decrease of selectivity may occur due to habituation to the setup and learning that actual mating is not possible. Further, the time to establish a stable preference might change depending on the number of choices given. In a comparable mate choice experiment that was also conducted in a four-choice setup lasting only 10 min, not all of the focal mice visited all possible partners before the end of the experiment [53]. In such short experiments, it is thus possible that mice had no chance to aquire all information necessary to make an informed decision. Thus we suggest to run pilot experiments with an extended period of time to deterime the duration most suitable to answer the question asked.

### Association between sexes – a measure for mate choice?

House mice live in socially sub-structured populations and form small reproductive units. In such units, one dominant male usually sires most of the offspring with one or several females [54, 55]. Dominant males defend their territories by frequent urinary marking and fighting with intruders [55–58]. It was shown, however, that not just male territories are important in the social structure of wild mice, but also the membership of males in a family group [31]. Montero and colleagues further observed that males sometimes shared nests with females and found repeated matings with the same partner within the same nest box (multiple nest boxes were offered in a large semi-natural setup). They suggest that familiarity is an important factor in repeated mating with the same partner [31].

We used a controlled cage setup to balance between the advantage of allowing sensory cues (which mice could use for individual assessment and potential mate choice [16, 46]) and the aim of not producing unwanted offspring for ethical reasons. It has been shown that social preferences measured in lab experiments do not necessarily lead to a higher number of matings between social partners [50, 51, 59, 60]. Thus, social preference cannot always be interpreted as mate choice. However, in the wild, there is usually not a direct choice between two or more possible partners like in a laboratory experiment. Under natural conditions, we expect selective attention, resulting in a higher likelihood to locate a preferred type of partner. This can eventually lead to a higher chance of mating.

Following these considerations, the behaviour observed (i.e. individuals of different sex spending time close to each other) can be an important step in mate choice and does not simply reflect a general social preference. Spending time with a potential partner not only increases the likelihood of mating with this partner (as a result of the increased chances to do so), but might also be interpreted as a form of securing a social resource.

### Selectivity depends on sex and population background

A clear difference in the strength of preference could be observed between pure and mixed individuals, with pure individuals being more selective. This supports the idea that homozygous individuals show stronger selectivity than heterozygous individuals, which was described previously by Yamazaki et al. [20]. Furthermore, we found a difference in the preference behaviour between females and males, which was mostly apparent during the first half of the experiment. Females generally show higher selectivity and stability than males. Stronger selectivity in female individuals compared to males is in line with hypotheses considering higher female investment in producing and raising offspring in species in which paternal care is not common [61].

The mate choice of males is supposedly driven by two strategies: either choosing one individual female and providing paternal care, or mating with as many females as possible without further commitments [62]. While the males in our study indeed had lower selectivity than females, this tendency was not significant when taking the average over the whole experimental period. Furthermore, males also have costs in reproduction, even in species with no paternal care, so the chance of having more viable offspring can be increased if males do not just arbitrarily mate with several females but make choices.

Over the whole experiment, the two sexes differed reciprocally in the strength of preference, depending on their fathers’ population background: males with a father from the French population and females with a father from the German population showed a stronger preference than the other two groups. We have no obvious explanation for such an interactive effect on selectivity depending not only on the sex but also the population background. However, this effect can at least partially be explained by the fact that the only factor correlating with the preference behaviour, i.e. MHCpat, was strongest in female mice with a father from the German population.

### Paternal influences on mate preference

In the mate choice experiment by Montero et al. [31], hybrid mice preferred paternally matching mates. Furthermore, assortative mating could be detected when mice had the chance to become familiar with individuals of their own population first. No assortative mating was observed when mice were unfamiliar with their own population before encountering individuals of the other population. In our experiments, all mice were unfamiliar, and like Montero et al. [31], we did not find assortative mating. Concerning paternal versus maternal matching, we found a stronger effect for paternal matching, but both values were not significant (Table 2). This suggests that there was not enough power in the experiment to resolve this issue.

Nevertheless, our results reveal another non-random partner preference in the form of a stronger preference for partners in which the MHC (H2-Eß locus) of the focal individual’s fathers and the MHC of the chosen satellite mouse show increased dissimilarity. This effect was only apparent in females with a father from the German population. In other words, female mice with a father from the German population spent more time with partners whose MHC locus was more different from that of the focal individual’s father than with those that had a smaller difference. This might be due to the higher mean dissimilarity between the paternal MHC of these mice and their satellite individuals’ MHC. We cannot exclude a similar effect for the other groups (females with a father from the French population and males independent of their father’s population) if more dissimilar individuals had been available.

There are two hypotheses regarding why MHC-driven mate choice might occur (selected reviews: [26, 63, 64]): the good genes hypothesis [65, 66], which corresponds to a partner preference based on MHC-diversity, and the complementary allele hypothesis [7, 67], which corresponds to a partner preference based on MHC-dissimilarity. Our findings support the complementary allele hypothesis for two reasons. First, we detected an effect of MHC-dissimilarity, expected under the complementary allele hypothesis, and not MHC-diversity, which would be more likely under the good gene hypothesis. Second, a detailed look at the haplotype distribution of the most and second most preferred individuals vs. never preferred individuals showed that neither individuals within the preferred individuals, nor within the non preferred individuals share certain haplotypes. Preferred and unpreferred individuals even share haplotypes. Thus, we found no evidence for certain MHC-alleles being favoured.

Several studies support mate choice based on MHC-complementary alleles, even though complementary alleles may include different degrees of dissimilarity (e.g. [68–70]). The effect of MHC-dissimilarity detected in our study is based on the MHC of the focal individual’s father. This effect does not involve self-referencing the individual’s own MHC during partner choice. It rather includes the information of the father’s MHC, which can be transmitted by either genomic or familial (sexual) imprinting from the father to its offspring in the first days in the nest while the father is still present (as shown by Montero et al. [31]).

Isles and colleagues support the hypothesis that preference behaviour is driven by genomic imprinting [49, 71]. They suggest that genes inherited with a parental bias are in linkage disequilibrium with odour determination and odorous information processing (e.g. MHC). There is no evidence that MHC genes themselves are inherited in a parent-of-origin manner (i.e. genomic imprinting [72]). The effects of familial imprinting and early learning are well known [73, 74]. Familial imprinting has been described to play a role in various situations, like kin recognition [75], mate choice [76, 77], and recognition of environmental cues [76, 78].

Penn and Potts [23] and Yamazaki et al. [25] have already suggested evidence of familial (olfactory) imprinting in mice. We thus propose that olfactory imprinting (i.e. early learning of the fathers smell) during the early days of life might be the mechanism underlying the fathers’ influence on their offspring’s preference behaviour in our study. It has already been described that familial imprinting takes place in the early nest phase [79–81]. Tramm and Servedio [82] suggest that paternally driven familial imprinting is more likely to evolve than maternal influences on mate choice. Furthermore, paternally driven familial imprinting might be a compromise between the two strategies of male mating behaviour described above (one partner, few offspring, and parental care vs. several partners, many offspring, and no parental care). Olfactory imprinting of MHC-information would enable the father to provide some care for all of its offspring (from several females) without being bound to a single nest.

## Conclusions

This study revealed two patterns of paternal influence on partner choice in wild mice: First, paternal population background in interaction with sex of the focal mouse had an influence on selecitivity, i.e. strength of preference. Second, the preference for a certain partner seems to be driven by the distance between the paternal MHC to the satellite mouse’ MHC, an effect we only observed in the group with the strongest preference, females with a German father. As MHC genes are not expressed in a parent-of-origin manner (genetic imprinting) we support the hypothesis of familial (olfactory) imprinting as most probable process for information transfer from father to offspring during the early days in the nest. Further studies under more natural conditions are needed to elucidate this process, with social interactions such as territoriality or the hierarchy structure of competing individuals taken into account. Finally, we wish to highlight the importance of an appropriate duration of behavioural experiments as the formation of preference is based on a real decision making process.

## Additional files

Additional file 1: Table S1. All individual information (microsatellite data, experimental information). (CSV 27 kb)

Additional file 2: Figure S1. Two examples visualising how to assess the consistency of choice (upper panel the “German” male “Hermann” and lower panel the “French” male “Jacques”). Two measures were used to define the consistency of choice. 1. Block-Count and 2. Block Size. Preference is based on the selectivity index SI calculated in intervals increasing by 10 minutes (the first interval being 10 minutes, the second 20 minutes, and so forth). For each time point the preferred cage (1-4) is plotted. “Hermann” changed his preference over time 4 times (= 5 blocks), compared to “Jacques” who changed his mind only once (= 2 blocks). After 38% of “Hermann” being in the experiment (as the last block is 62%), he chose the mouse from cage 4 to be his preferred partner, “Jacques” already decided after just 5% of the total time in the experiment that mouse 1 is the best. “Hermann” never chose mouse 3 and “Jacques” never chose mice 1 and 2. (PDF 35 kb)

Additional file 3: Figure S2. a) Allele sharing tree (Bowcock *et al.* 1994) for all animals based on microsatellites. Even though a separation between populations is evident, small branch lengths reflect a still close relationship between individuals of all breeding types. b) Neighbour-joining tree of H2-Eß locus Exon 2 haplotype sequences. Bootstrap values > 50 are shown. No pattern of population divergence can be detected, both populations share several alleles. (PNG 301 kb)

Additional file 4: Figure S3. Mouse activity patterns over the whole experimental period and diurnal rhythm of activity averaged over all days. a) Average activity measured as number of antenna hits per hour for each day of the experiment. After a steep drop of activity in the beginning of the experiment, activity steadily drops unitl the end. Boxplots include outliers (black dots) and additionally all individual data points (grey dots, slightly jittered along the x-axis for clarity). b) Diurnal rhythm of mouse activity. Each light grey line shows the number of antenna hits per hour of individual as a proxy for its activity. The open circle represent the average over all mice of the given time of the day. The light yellow box indicates the phase of lights-on in the experimental room. Mice clearly are active all over the day, with a siesta in the early afternoon (12:00 – 15:00h). (PDF 618 kb)

## Abbreviations

CAS: Cavalli-Sforza-distance between a satellite mouse and the focal individual
CASmat: Cavalli-Sforza-distance between a satellite mouse and the focal individual’s mother
CASpat: Cavalli-Sforza-distance between a satellite mouse and the focal individual’s father
F: French population
FF: Mouse with pure French population background
FG: Mouse with a mother from the French and a father from the German population
G: German population
GF: Mouse with a mother from the German and a father from the French population
GG: Mouse with pure German population background
LRT: Likelihood Ratio Test
MHC: p-distance between a satellite mouse and the focal individual
MHCmat: p-distance between a satellite mouse and the focal individual’s mother
MHCpat: p-distance between a satellite mouse and the focal individual’s father
SI: Selectivity index
xF: Mouse with a father from the French population and a mother from any of the two populations
xG: Mouse with a father from the German population and a mother from any of the two populations

## References

[1] Kokko H, Brooks R, Jennions MD, Morley J (2003). The evolution of mate choice and mating biases. Proc R Soc Lond B Biol Sci.

[2] Andersson M, Simmons LW (2006). Sexual selection and mate choice. Trends Ecol Evol.

[3] Sardell RJ, Kempenaers B, DuVal EH (2014). Female mating preferences and offspring survival: testing hypotheses on the genetic basis of mate choice in a wild lekking bird. Mol Ecol.

[4] Ihle M, Kempenaers B, Forstmeier W (2015). Fitness benefits of mate choice for compatibility in a socially monogamous species. PLoS Biol.

[5] Raveh S, Sutalo S, Thonhauser KE, Thoß M, Hettyey A, Winkelser F, Penn DJ (2014). Female partner preferences enhance offspring ability to survive an infection. BMC Evol Biol.

[6] Drickamer LC, Gowaty PA, Holmes CM (2000). Free female mate choice in house mice affects reproductive success and offspring viability and performance. Anim Behav.

[7] Tregenza T, Wedell N (2000). Genetic compatibility, mate choice and patterns of parentage: invited review. Mol Ecol.

[8] Dieckmann U, Metz JAJ, Doebeli M, Tautz D (2004). Adaptive Speciation.

[9] Crow JF, Kimura M (1970). An introduction to population genetics theory.

[10] Lewontin RC (1974). The genetic basis of evolutionary change.

[11] Jiang Y, Bolnick DI, Kirkpatrick M (2013). Assortative mating in animals. Am Nat.

[12] Burley N (1983). The meaning of assortative mating. Ethol Sociobiol.

[13] Sherborne AL, Thom MD, Paterson S, Jury F, Ollier WE, Stockley P, Beynon RJ, Hurst JL (2007). The genetic basis of inbreeding avoidance in house mice. Curr Biol.

[14] Mucignat-Caretta C, Caretta A (2014). Message in a bottle: major urinary proteins and their multiple roles in mouse intraspecific chemical communication. Anim Behav.

[15] Leinders-Zufall T, Brennan P, Widmayer P, Maul-Pavicic A, Jäger M, Li X-H, Breer H, Zufall F, Boehm T (2004). MHC class I peptides as chemosensory signals in the vomeronasal organ. Science.

[16] Asaba A, Hattori T, Mogi K, Kikusui T (2014). Sexual attractiveness of male chemicals and vocalizations in mice. Front Neurosci.

[17] Holy TE, Guo Z (2005). Ultrasonic songs of male mice. PLoS Biol.

[18] Hoffmann F, Musolf K, Penn DJ (2012). Ultrasonic courtship vocalizations in wild house mice: spectrographic analyses. J Ethol.

[19] Klein J (1979). The major histocompatibility complex of the mouse. Science.

[20] Yamazaki K, Boyse E, Mike V, Thaler H, Mathieson B, Abbott J, Boyse J, Zayas Z, Thomas L (1976). Control of mating preferences in mice by genes in the major histocompatibility complex. J Exp Med.

[21] Ziegler A, Kentenich H, Uchanska-Ziegler B (2005). Female choice and the MHC. Trends Immunol.

[22] Milinski M (2006). The major histocompatibility complex, sexual selection, and mate choice. Annu Rev Ecol Evol Syst.

[23] Penn DJ, Potts WK (1999). The evolution of mating preferences and major histocompatibility complex genes. Am Nat.

[24] Penn D, Musolf K, Machol An M, Baird SJE, Munclinger P, Pi Alek J (2012). The evolution of MHC diversity in house mice. Evolution of the house mouse.

[25] Yamazaki K, Beauchamp GK, Kupniewski D, Bard J, Thomas L, Boyse E (1988). Familial imprinting determines H-2 selective mating preferences. Science.

[26] Immelmann K. Ecological significance of imprinting and early learning. Annu Rev Ecol Syst. 1975;6:15–37.

[27] Teschke M, Mukabayire O, Wiehe T, Tautz D (2008). Identification of selective sweeps in closely related populations of the house mouse based on microsatellite scans. Genetics.

[28] Ihle S, Ravaoarimanana I, Thomas M, Tautz D (2006). An analysis of signatures of selective sweeps in natural populations of the house mouse. Mol Biol Evol.

[29] Staubach F, Lorenc A, Messer PW, Tang K, Petrov DA, Tautz D (2012). Genome patterns of selection and introgression of haplotypes in natural populations of the house mouse (*Mus musculus*). PLoS Genet.

[30] von Merten S, Hoier S, Pfeifle C, Tautz D (2014). A role for ultrasonic vocalisation in social communication and divergence of natural populations of the house mouse (Mus musculus domesticus). PLoS One.

[31] Montero I, Teschke M, Tautz D (2013). Paternal imprinting of mating preferences between natural populations of house mice (*Mus musculus domesticus*). Mol Ecol.

[32] Bingel AS, Schwartz NB (1969). Pituitary LH content and reproductive tract changes during the mouse oestrous cycle. J Reprod Fertil.

[33] Whitten WK (1956). Modification of the oestrous cycle of the mouse by external stimuli associated with the male. J Endocrinol.

[34] R Core Team (2016). R: A Language and Environment for Statistical Computing.

[35] Bowcock A, Ruiz-Linares A, Tomfohrde J, Minch E, Kidd J, Cavalli-Sforza LL (1994). High resolution of human evolutionary trees with polymorphic microsatellites. Nature.

[36] Dieringer D, Schlötterer C (2003). Microsatellite analyser (MSA): a platform independent analysis tool for large microsatellite data sets. Mol Ecol Notes.

[37] Tamura K, Stecher G, Peterson D, Filipski A, Kumar S (2013). MEGA6: molecular evolutionary genetics analysis version 6.0. Mol Biol Evol.

[38] Thompson JD, Higgins DG, Gibson TJ (1994). CLUSTAL W: improving the sensitivity of progressive multiple sequence alignment through sequence weighting, position-specific gap penalties and weight matrix choice. Nucleic Acids Res.

[39] Stephens M, Donnelly P (2003). Ancestral inference in population genetics models with selection (with discussion). Aust N Z J Stat.

[40] Stephens M, Smith NJ, Donnelly P (2001). A new statistical method for haplotype reconstruction from population data. Am J Hum Genet.

[41] Rozas J. DNA Sequence Polymorphism Analysis using DnaSP. In Posada, D. (ed.) Bioinformatics for DNA Sequence Analysis; Methods in Molecular Biology Series Vol. 537. NJ, USA: Humana Press; 2009. p. 337–350. 10.1007/978-1-59745-251-9_17 19378153

[42] Dallmann R, Mrosovsky N (2006). Scheduled wheel access during daytime: A method for studying conflicting zeitgebers. Physiol Behav.

[43] Aschoff J. Circadian system properties. In Environmental Physiology 1981: Proceedings of the 28th International Congress of Physiological Sciences, Budapest, 1980. pp 1–17.

[44] de Groot MH, Rusak B (2004). Housing conditions influence the expression of food-anticipatory activity in mice. Physiol Behav.

[45] de Visser L, van den Bos R, Spruijt BM (2005). Automated home cage observations as a tool to measure the effects of wheel running on cage floor locomotion. Behav Brain Res.

[46] Musolf K, Hoffmann F, Penn DJ (2010). Ultrasonic courtship vocalizations in wild house mice, Mus musculus musculus. Anim Behav.

[47] Latour Y, Perriat-Sanguinet M, Caminade P, Boursot P, Smadja CM, Ganem G (2014). Sexual selection against natural hybrids may contribute to reinforcement in a house mouse hybrid zone. Proc R Soc Lond B Biol Sci.

[48] Asaba A, Okabe S, Nagasawa M, Kato M, Koshida N, Osakada T, Mogi K, Kikusui T (2014). Developmental social environment imprints female preference for male song in mice. PLoS One.

[49] Isles AR, Baum MJ, Ma D, Keverne EB, Allen ND (2001). Genetic imprinting: urinary odour preferences in mice. Nature.

[50] Thonhauser KE, Raveh S, Hettyey A, Beissmann H, Penn DJ (2013). Scent marking increases male reproductive success in wild house mice. Anim Behav.

[51] Manser A, König B, Lindholm AK (2015). Female house mice avoid fertilization by t haplotype incompatible males in a mate choice experiment. J Evol Biol.

[52] Whitten WK (1959). Occurrence of anoestrus in mice caged in groups. J Endocrinol.

[53] Roberts SC, Gosling LM (2003). Genetic similarity and quality interact in mate choice decisions by female mice. Nat Genet.

[54] Crowcroft P, Rowe FP. Social organization and territorial behaviour in the wild house mouse (*Mus musculus* L.). In Proceedings of the Zoological Society of London. London; 1963;(140):517–31.

[55] Reimer J, Petras M (1967). Breeding structure of the house mouse, Mus musculus, in a population cage. J Mammal.

[56] Poole T, Morgan H (1975). Aggressive behaviour of male mice (Mus musculus) towards familiar and unfamiliar opponents. Anim Behav.

[57] Poole TB, Morgan H (1976). Social and territorial behaviour of laboratory mice (Mus musculus L.) in small complex areas. Anim Behav.

[58] Wolff RJ (1985). Mating behaviour and female choice: their relation to social structure in wild caught House mice (Mus musculus) housed in a semi-natural environment. J Zool.

[59] Zala SM, Bilak A, Perkins M, Potts WK, Penn DJ (2015). Female house mice initially shun infected males, but do not avoid mating with them. Behav Ecol Sociobiol.

[60] Rolland C, MacDonald DW, De Fraipont M, Berdoy M (2003). Free female choice in house mice: leaving best for last. Behaviour.

[61] Trivers R. Parental investment and sexual selection In Sexual selection and the descent of man edited by B. Campbell, 1871–1971. Chicago, Illinois: Aldine Press; 1972.

[62] Clutton-Brock TH (1989). Review lecture: mammalian mating systems. Proc R Soc Lond B Biol Sci.

[63] Mays HL, Hill GE. Choosing mates: good genes versus genes that are a good fit. Trends Ecol Evol. 2004;19(10):554–9. 10.1016/j.tree.2004.07.018 16701321

[64] Piertney S, Oliver M (2006). The evolutionary ecology of the major histocompatibility complex. Heredity.

[65] Kamiya T, O'Dwyer K, Westerdahl H, Senior A, Nakagawa S (2014). A quantitative review of MHC-based mating preference: the role of diversity and dissimilarity. Mol Ecol.

[66] Von Schantz T, Wittzell H, Goransson G, Grahn M, Persson K (1996). MHC genotype and male ornamentation: genetic evidence for the Hamilton-Zuk model. Proc R Soc Lond B Biol Sci.

[67] Hamilton WD, Zuk M (1982). Heritable true fitness and bright birds: a role for parasites?. Science.

[68] Zeh JA, Zeh DW (1996). The evolution of polyandry I: intragenomic conflict and genetic incompatibility. Proc R Soc Lond B Biol Sci.

[69] Huchard E, Baniel A, Schliehe-Diecks S, Kappeler PM (2013). MHC-disassortative mate choice and inbreeding avoidance in a solitary primate. Mol Ecol.

[70] Jacob S, McClintock MK, Zelano B, Ober C (2002). Paternally inherited HLA alleles are associated with women's choice of male odor. Nat Genet.

[71] Reusch TB, Häberli MA, Aeschlimann PB, Milinski M (2001). Female sticklebacks count alleles in a strategy of sexual selection explaining MHC polymorphism. Nature.

[72] Isles AR, Baum MJ, Ma D, Szeto A, Keverne EB, Allen ND (2002). A possible role for imprinted genes in inbreeding avoidance and dispersal from the natal area in mice. Proc R Soc Lond B Biol Sci.

[73] Lorenc A, Linnenbrink M, Montero I, Schilhabel MB, Tautz D. Genetic differentiation of hypothalamus parentally biased transcripts in populations of the house mouse implicate the Prader-Willi syndrome imprinted region as a possible source of behavioral divergence. Mol Biol Evol. 2014;31:3240–9. 10.1093/molbev/msu257 PMC4245819 25172960

[74] Svensson EI, Eroukhmanoff F, Karlsson K, Runemark A, Brodin A (2010). A role for learning in population divergence of mate preferences. Evolution.

[75] Gerlach G, Hodgins-Davis A, Avolio C, Schunter C (2008). Kin recognition in zebrafish: a 24-hour window for olfactory imprinting. Proc R Soc Lond B Biol Sci.

[76] Caspers BA, Hoffman JI, Kohlmeier P, Krüger O, Krause ET (2013). Olfactory imprinting as a mechanism for nest odour recognition in zebra finches. Anim Behav.

[77] Milinski M, Griffiths S, Wegner KM, Reusch TB, Haas-Assenbaum A, Boehm T (2005). Mate choice decisions of stickleback females predictably modified by MHC peptide ligands. Proc Natl Acad Sci U S A.

[78] Hinz C, Namekawa I, Behrmann-Godel J, Oppelt C, Jaeschke A, Müller A, Friedrich RW, Gerlach G. Olfactory imprinting is triggered by MHC peptide ligands. Sci Rep. 2013;3:1–8. 10.1038/srep02800 PMC3786304 24077566

[79] Fillion TJ, Blass EM (1986). Infantile experience with suckling odors determines adult sexual behavior in male rats. Science.

[80] D'Udine B, Alleva E. Early experience and sexual preferences in rodents. In P. Bateson, eds. Mate choice. Cambridge: Cambridge University Press; 1983. p. 311–27.

[81] Leon M (1983). Chemical communication in mother-young interactions. Pheromones Reprod Mammals.

[82] Tramm NA, Servedio MR (2008). Evolution of mate-choice imprinting: competing strategies. Evolution.

