# Supplementary material for: No speed dating please! Patterns of social preference in male and female house mice
Source: Front Zool. 2017 Jul 24;14:38. doi: 10.1186/s12983-017-0224-y (PMC5525247; doi:10.1186/s12983-017-0224-y)

Determination of Consistency of Choice:

1: Block-Count 2: Size of the last block [relative to the total amount with any satellite mouse]

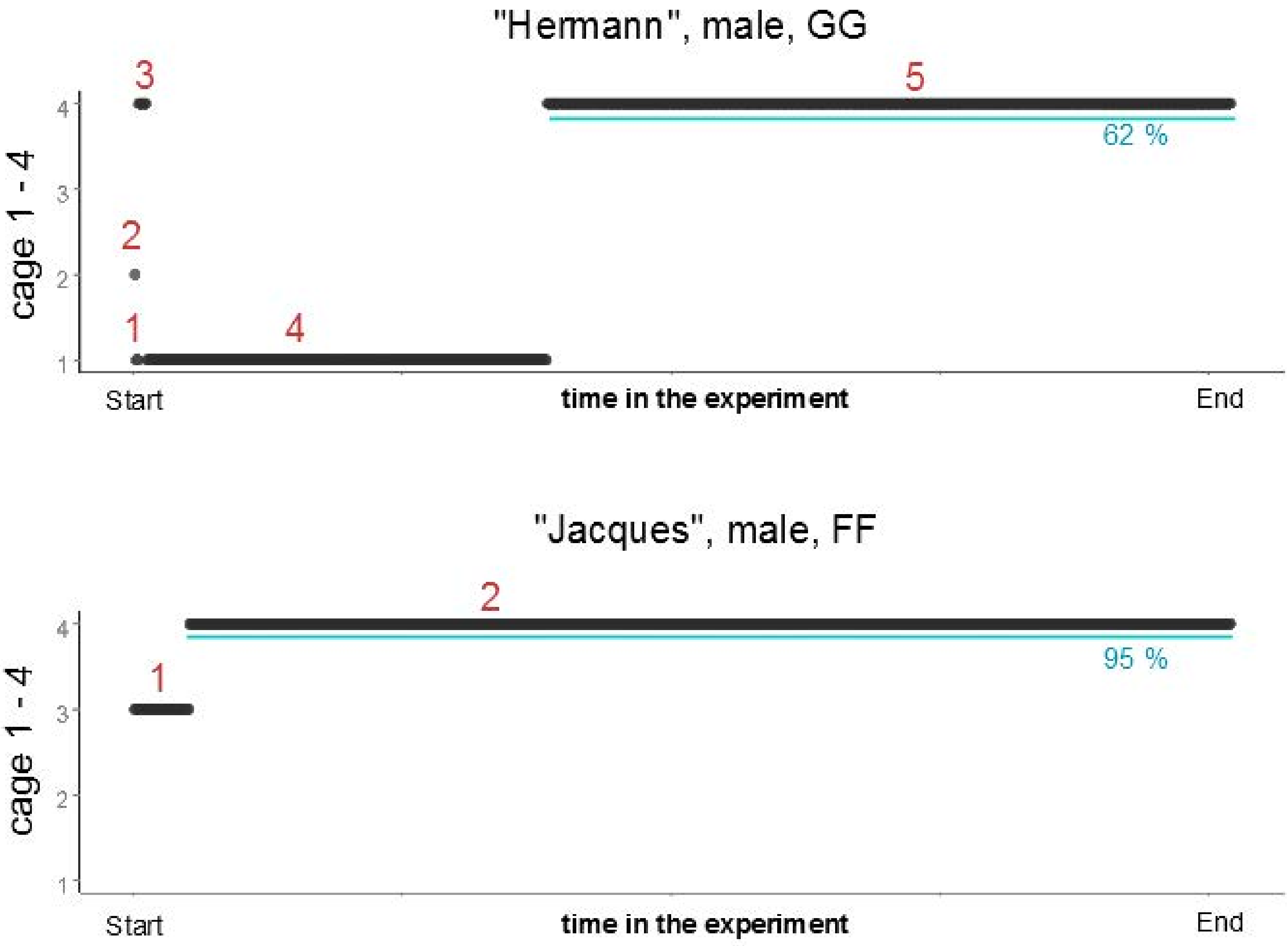

Supplement: Supplementary file 2 — Two examples visualising how to assess the consistency of choice (upper panel the “German” male “Hermann” and lower panel the “French” male “Jacques”). Two measures were used to define the consistency of choice. 1. Block-Count and 2. Block Size. Preference is based on the selectivity index SI calculated in intervals increasing by 10 minutes (the first interval being 10 minutes, the second 20 minutes, and so forth). For each time point the preferred cage (1-4) is plotted. “Hermann” changed his preference over time 4 times (= 5 blocks), compared to “Jacques” who changed his mind only once (= 2 blocks). After 38% of “Hermann” being in the experiment (as the last block is 62%), he chose the mouse from cage 4 to be his preferred partner, “Jacques” already decided after just 5% of the total time in the experiment that mouse 1 is the best. “Hermann” never chose mouse 3 and “Jacques” never chose mice 1 and 2. (PDF 35 kb) [file 12983_2017_224_MOESM2_ESM.pdf]
