# Supplementary figures and images for: No speed dating please! Patterns of social preference in male and female house mice
Source: Front Zool. 2017 Jul 24;14:38. doi: 10.1186/s12983-017-0224-y (PMC5525247; doi:10.1186/s12983-017-0224-y)

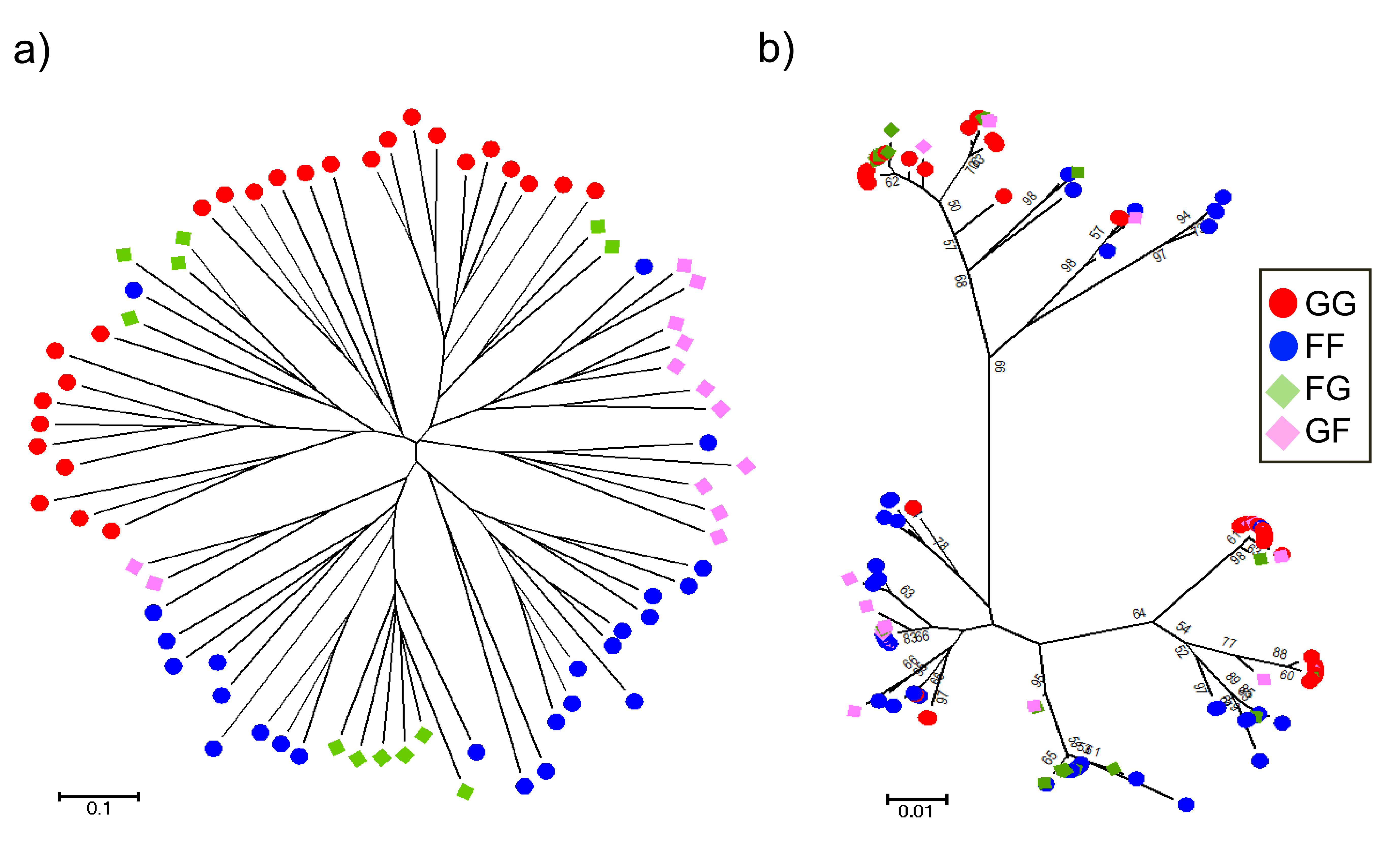

Supplement: Supplementary file 3 — a) Allele sharing tree (Bowcock et al. 1994) for all animals based on microsatellites. Even though a separation between populations is evident, small branch lengths reflect a still close relationship between individuals of all breeding types. b) Neighbour-joining tree of H2-Eß locus Exon 2 haplotype sequences. Bootstrap values > 50 are shown. No pattern of population divergence can be detected, both populations share several alleles. (PNG 301 kb) [file 12983_2017_224_MOESM3_ESM.png]

a)

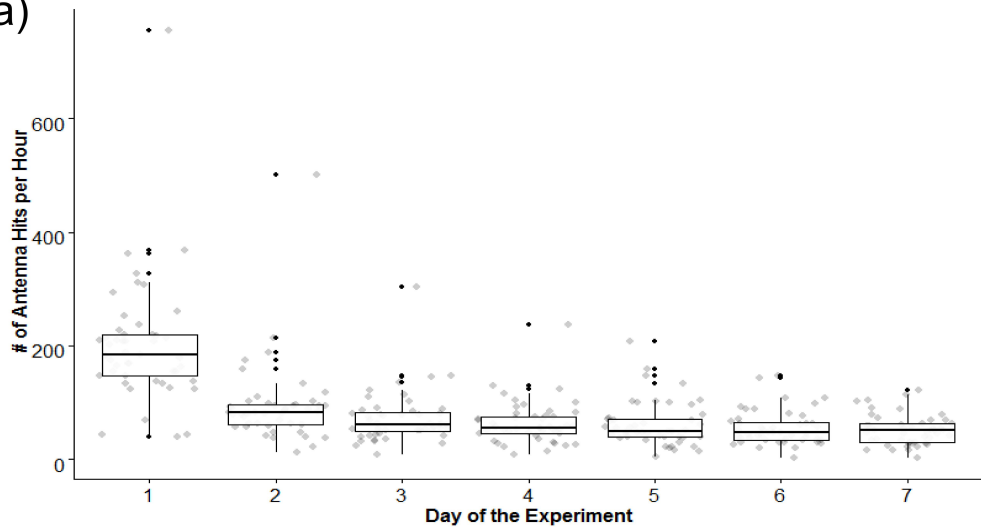

b)

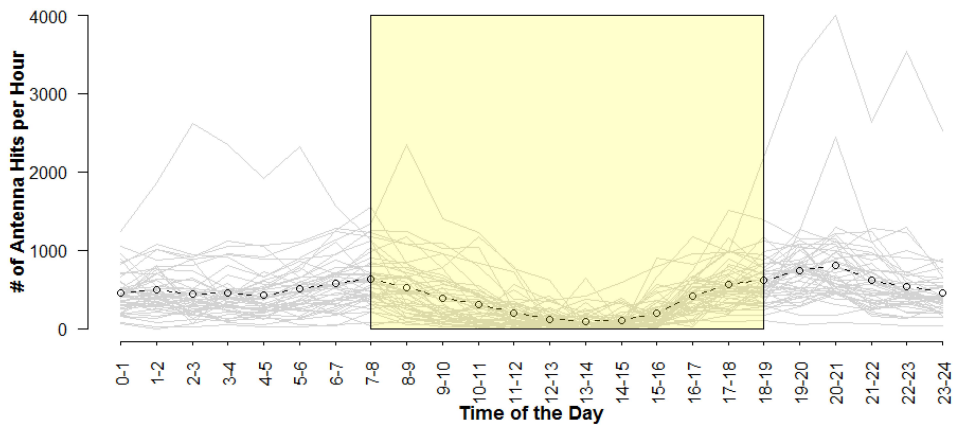

Supplement: Supplementary file 4 — Mouse activity patterns over the whole experimental period and diurnal rhythm of activity averaged over all days. a) Average activity measured as number of antenna hits per hour for each day of the experiment. After a steep drop of activity in the beginning of the experiment, activity steadily drops unitl the end. Boxplots include outliers (black dots) and additionally all individual data points (grey dots, slightly jittered along the x-axis for clarity). b) Diurnal rhythm of mouse activity. Each light grey line shows the number of antenna hits per hour of individual as a proxy for its activity. The open circle represent the average over all mice of the given time of the day. The light yellow box indicates the phase of lights-on in the experimental room. Mice clearly are active all over the day, with a siesta in the early afternoon (12:00 – 15:00h). (PDF 618 kb) [file 12983_2017_224_MOESM4_ESM.pdf]
